# Synthesis and Surface Activity of Cationic Amino Acid-Based Surfactants in Aqueous Solution

**DOI:** 10.1007/s11743-017-2002-4

**Published:** 2017-07-31

**Authors:** Katarzyna E. Greber

**Affiliations:** Department of Physical Chemistry, Faculty of Pharmacy, Medical University of Gdansk, Gdansk, Poland

**Keywords:** Lipopeptides, Amino acid-based surfactants, CMC, Surface tension, Emulsion stability

## Abstract

I studied the possibility of using amino acid-based surfactants as emulsifiers at the same time as preservatives. Fourteen lipopeptides were synthesized employing a solid phase peptide synthesis procedure. All compounds were designed to be positively charged from +1 to +4 and acylated with fatty acid chain—palmitic and miristic. The surface activity of the obtained lipopeptides was tested using a semi-automatic tensiometer to calculate parameters describing the behavior of lipopeptides in the air/water interface. Such parameters as CMC, surface tension at the CMC point (*σ*_CMC_), effectiveness (*π*_CMC_), and efficiency (pC20) were measured. Emulsifying properties of all lipopeptides were also examined. The studies reveal that the surface active properties of synthesized compounds strongly depend on the length of alkyl chains as well as on the composition of amino acid polar heads. The critical micelle concentration decreases with increasing alkyl chain length of lipopeptides with the same polar head. The effectiveness and efficiency decrease when the number of amino acids in the polar head increases. All lipopeptides established a very weak emulsification power and created unstable water/Miglyol 812 and water/paraffin oil emulsions. Results suggest that lipopeptides cannot be used as emulsifiers; nonetheless, it is possible to use them as auxiliary surfactants with disinfectant properties in combination with more potent emulsifiers.

## Introduction

Biosurfactants are a large class of naturally occurring compounds produced by numerous bacteria, yeasts and fungi. They can be divided into several groups, e.g. rhamnolipids, glycolipids, lipopolysaccharides and lipopeptides [1–4]. Lipopeptides consist of an amino acid polar head and lipophilic tail, which is mostly formed by a fatty acid chain. In recent years, due to their high commercialization potential, this group of compounds has been widely studied and their biological and physicochemical properties precisely described [5–8].

Some naturally occurring lipopeptides, e.g. daptomycin, are already commercially available. Daptomycin is a lipopeptide antibiotic with activity toward most strains of staphylococci and streptococci. It is administered when methicyllin-resistant *Staphylococcus aureus* or vancomycin-resistant *Enterococcus* infection occurs [9, 10]. A further example of a commercially available lipopeptide with surfactant-like structure is biologically active polymyxin produced by Gram-positive bacteria—*Paenibacillus polymyxa*. This lipopeptide antibiotic is currently the last-line therapy of multidrug-resistant Gram-negative bacteria infections [11].

Surfactin is a powerful natural lipopeptide surfactant. It is produced by various strains of the gram-positive, endospore-producing *Bacillus subtilis*. It has excellent surface active properties and proven anti-bacterial, anti-viral, anti-fungal and anti-mycoplasma activity [12–14]. Biosurfactants can also be exploited in environmental applications such as bioremediation. The surface-active lipopeptides are able to increase the surface area of water-insoluble organic pollutants and thus increase the rate of their degradation by enhancing the growth of microorganisms. There are also advanced ongoing studies on the application of biosurfactants in the oil recovery industry [7, 15–17].

Aminat-G is a personal care and cosmetic product containing cationic surfactant ethyl-Nα-dodecanoyl-l-arginate hydrochloride (LAE), which exhibits antimicrobial activity. In September 2005, LAE was classified by the US Food and Drug Administration (FDA) as a substance safe for use in food. Due to its antimicrobial activity, biodegradability, low toxicity to aquatic organisms and lack of irritation to eyes and skin at the recommended concentration, LAE has been approved by the European Union as a preservative. In addition, LAE is a possible ingredient for use in anti-dandruff shampoos, deodorants and antibacterial soaps [18].

The aim of this study was to obtain, via chemical synthesis, a group of cationic lipopeptides of simple alkyl chains, palmitoyl (Palm) and myristoyl (Myr), which would simultaneously fulfill the roles of preservative and emulsifier. All compounds were designed, similarly to Aminat-G, to possess amphipathic structures and positive charge, the features responsible for surface active properties and antimicrobial activity. Fourteen cationic lipopeptides, with already tested antimicrobial activity [19], (Tables 1, 2) were synthesized. Their surface-active and emulsifying properties are characterized in this paper. Due to their chemical similarity to LAE, some of these substances could be potentially employed as new cosmetic antimicrobials.

**Table 1:** Selected parameters characterizing synthesized lipopeptide surfactants acylated with palmitic fatty acid

No.	Lipopeptide	Net charge	CMC		pC_20_	*σ*_CMC_ (mN/m)	*π*_CMC_ (mN/m)	Emulsion stability (s)
			(mmol/L)	(μg/mL)				
1.	Palm-K-NH_2_	+1	0.29	111.2	3.40	36.5	35.0	44
2.	Palm-KK-NH_2_	+2	1.07	547.60	3.23	45.1	26.4	62
3.	Palm-KKK-NH_2_	+3	4.8	3.07 × 10^3^	2.41	49.9	21.6	47
4.	Palm-KKKK-NH_2_	+4	14.6	11.2 × 10^3^	1.88	50.9	20.6	42
5.	Palm-KG-NH_2_	+1	0.29	127.80	3.95	40.8	30.7	40
6.	Palm-KGK-NH_2_	+2	1.89	1.07 × 10^3^	2.90	47.1	24.4	44
7.	Palm-KGKG-NH_2_	+2	3.70	2.3 × 10^3^	2.55	47.6	23.9	50

**Table 2:** Selected parameters characterizing synthesized lipopeptide surfactants acylated with myristic fatty acid

No.	Lipopeptide	Net charge	CMC		pC_20_	*σ*_CMC_ (mN/m)	*π*_CMC_ (mN/m)	Emulsion stability (s)
			(mmol/L)	(μg/mL)				
8.	Myr-K-NH_2_	+1	1.02	362.66	3.64	38.1	33.4	42
9.	Myr-KK-NH_2_	+2	4.40	2.13 × 10^3^	2.52	46.6	24.9	55
10.	Myr-KKK-NH_2_	+3	11.70	7.16 × 10^3^	2.00	49.9	21.6	44
11.	Myr-KKKK-NH_2_	+4	18.04	13.35 × 10^3^	1.81	50.4	21.1	40
12.	Myr-KG-NH_2_	+1	1.06	437.36	3.46	41.2	30.3	41
13.	Myr-KGK-NH_2_	+2	2.80	1.5 × 10^3^	2.77	46.0	25.5	46
14.	Myr-KGKG-NH_2_	+2	6.90	4.12 × 10^3^	2.28	48.0	23.5	51

## Experimental Procedures

### Lipopeptide Synthesis, Purification and Analysis

**Fig. 1:**
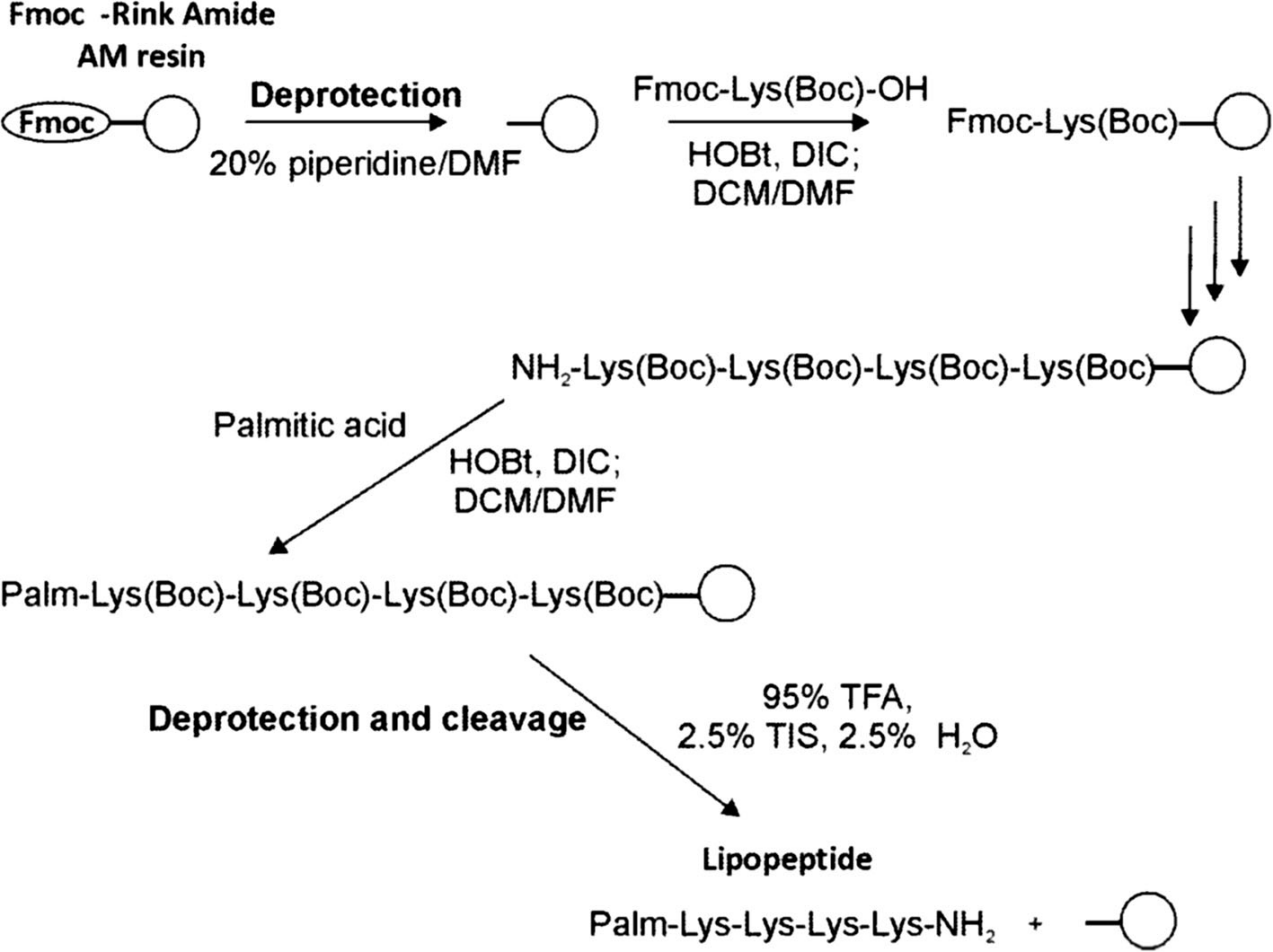
Synthesis of selected lipopeptide acylated with palmitic fatty acid

All reagents and chemicals were of analytical grade. Water was obtained from a Milli-Q system (Merck Millipore, Germany) equipped with a 0.22 μm filter and had a resistivity of >18.2 MΩ cm. Amino acid-based cationic surfactants were synthesized employing a solid-phase peptide synthesis (SPPS) procedure and the 9-fluorenylmethoxycrbonyl (Fmoc) methodology (Fig. 1) on Fmoc-Rink Amide AM resin (1.5 mmol/g, Iris Biotech, Germany). The following amino acid derivatives were used to assemble the amino acid fragments of the lipopeptides: lysine (Lys, K) with ε-amino group protected with *tert*-butyloxycarbonyl (Boc) group—Fmoc-Lys(Boc)-OH, and glycine (Gly, G)—Fmoc-Gly-OH. The Fmoc group was detached by a 20% solution of piperidine in *N*,*N*-dimethylformamide (DMF). Peptide bond formation was carried out with a two-fold excess of Fmoc-protected amino acid in a *N*,*N*-dimethylformamide/dichloromethane (DCM) mixture (1:1 v/v) with diisopropylocarbodiimide (DIC) and 1-hydroxybenzotriazole (HOBt). The reaction was monitored with the chloranil test. Cleavage from the resin was achieved by trifluoroacetic acid (TFA) with triisopropylsilane (TIS) and water (95/2.5/2.5) for 2 h. Cleaved lipopeptides were precipitated with cold diethyl ether and freeze-dried [20]. They were then purified via semi-preparative reverse-phase high performance liquid chromatography (RP-HPLC) on a Nucleodur C8 ec column, 10 × 250 mm, 100 Å (Macherey–Nagel, Germany). Synthesized compounds were eluted with a linear gradient 20–60% of phase B (phase A—0.1% TFA in water, phase B—0.1% TFA in acetonitrile), at a flow rate of 3 mL/min, at 214 nm. Purified lipopeptides were analyzed via RP-HPLC on a Chromolith Performance RP-18 (100 × 4.6 mm) monolithic column (Merck, Germany) with a linear gradient 2–98% of phase B, at a flow rate of 2 mL/min, at 214 nm. Fractions with purity greater than 95% were pooled and freeze-dried. Lipopeptides were further characterized and identified via matrix assisted laser desorption ionization time-of-flight mass spectrometry (MALDI TOF, Biflex III, Bruker, Germany) and Fourier transform infrared spectroscopy (FT/IR-4100, Jasco, Germany). Infrared spectra were recorded over the range 4000–800 cm^−1^ using the potassium bromide tablet method in the transmittance mode. Tablets were prepared from 100 mg of KBr and 1.5 mg of the tested lipopeptide.

#### Surface Active Parameters

The critical micelle concentration of each lipopeptide surfactant was determined from surface tension measurements performed on a semi-automatic EasyDyne (Kruss, Germany) tensiometer [21]. Solutions of known concentrations were progressively diluted and examined by the Du Nouy ring method. Temperature was maintained at 25 ± 0.2 °C using a thermostated water bath. The CMC values were calculated by plotting the surface tension against the log of the concentration of the lipopeptide surfactant. All measurements were conducted in unbuffered aqueous solutions. The accuracy of the surface tension measurements was 0.1 mN/m.

#### Emulsification Properties

The emulsification properties of the synthesized lipopeptides were measured by mixing 10 mL of surfactant solution **(**1%**)** in water and 5 mL of oily phase for 2 min, 300 rpm, at 20 °C in a homogenizer (SilentCrusher M, Heildolph, Germany). Two oily phases, paraffin oil and triglyceride (Miglyol 812), were employed to obtain two different emulsion systems. The emulsification power has been expressed as the time required for visible separation of 9 mL of water phase from the oily phase [22, 23].

## Results and Discussion

The potentiometric titration curve of lysine indicates that ε-amino group, in the wide pH range between 0 and 10.5, is protonated. The −log of dissociation constant of the ε-amino group of lysine does not change significantly under the influence of peptide bond formation, and for peptides and lipopeptides it is about 10 [24]. Since the ε-amino group of lysine, forming a hydrophilic head of tested lipopeptides, in the wide pH range of 0–10 is positively charged, the net positive charge of each lipopeptide is equal to the number of lysine residues incorporated in the hydrophilic head. It means that, in the conditions of the experiments performed here, all the lipopeptides were positively charged. The net charges of tested lipopeptides are summarized in Tables 1 and 2.

To confirm the identity of the synthesized compounds, the MALDI-TOF and FT/IR methods were used. The MALDI-TOF technique is characterized by mild ionization of the sample, therefore there was no fragmentation of tested substances. Thus the interpretation of mass spectra was confined to comparing the mass of molecular peak [M + H^+^] with the calculated molecular mass of the lipopeptide. All the lipopeptides obtained by SPPS corresponded to designed structures. Giacometti et al. [25] and Sarig et al. [26] also used the MALDI-TOF method of identifying synthetic peptide compounds and found it sufficient. Some research studies report the possibility of additional confirmation of the identity of peptide compounds by performing, e.g., amino acid analysis [27, 28]. FTIR spectroscopy was used to confirm the identity of the functional groups of synthesized lipopeptide surfactants. The FT/IR spectra of all lipopeptides showed the absorption bands at: 3303–3287 cm^−1^ corresponding to the presence of the –NH stretching vibrations, 2928–2848 cm^−1^ reflecting the stretch (–CH) of CH_2_ and CH_3_ groups in the aliphatic chains, 1679–1632 cm^−1^ corresponding to amide I (C=O) bonds, 1543–1529 cm^−1^ indicate the presence of amide II (N–H) bonds. Detailed data describing selected physicochemical properties of tested lipopeptides are presented below.

Lipopeptide 1: Palm-K-NH_2_; *N*-α-palmitoyl-l-lysine-amide, yield 85%, white solid. RP-HPLC retention factor* k* 8.17 (gradient B 2–98%/30 min); MS (MALDI-TOF) *m*/*z* 384.4 [M + H]^+^; MW (monois.) 383.4; FTIR: 3301, 2919, 2850, 1673, 1529 cm^−1^.

Lipopeptide 2: Palm-KK-NH_2_; *N*-α-palmitoyl-l-lysyl-l-lysine-amide, yield 87%, white solid. RP-HPLC retention factor* k* 7.02 (gradient B 2–98%/30 min); MS (MALDI-TOF) *m*/*z* 512.5 [M + H]^+^; MW (monois.) 511.5; FTIR: 3293, 2922, 2854, 1676, 1529 cm^−1^.

Lipopeptide 3: Palm-KKK-NH_2_; *N*-α-palmitoyl-l-lysyl-l-lysyl-l-lysine-amide, yield 75%, white solid. RP-HPLC retention factor* k* 6.58 (gradient B 2–98%/30 min); MS (MALDI-TOF) *m*/*z* 640.5 [M + H]^+^; MW (monois.) 639.5; FTIR: 3290, 2922, 2853, 1674, 1532 cm^−1^.

Lipopeptide 4: Palm-KKKK-NH_2_; *N*-α-palmitoyl-l-lysyl-l-lysyl-l-lysyl-l-lysine-amide, yield 70%, white solid. RP-HPLC retention factor* k* 6.20 (gradient B 2–98%/30 min); MS (MALDI-TOF) *m*/*z* 768.6 [M + H]^+^; MW (monois.) 767.6; FTIR: 3292, 2919, 2853, 1672, 1536 cm^−1^.

Lipopeptide 5: Palm-KG-NH_2_; *N*-α-palmitoyl-l-lysyl-l-glycine-amide, yield 85%, white solid. RP-HPLC retention factor* k* 8.24 (gradient B 2–98%/30 min); MS (MALDI-TOF) *m*/*z* 441.4 [M + H]^+^; MW (monois.) 440.4; FTIR: 3303, 2925, 2856, 1678, 1534 cm^−1^.

Lipopeptide 6: Palm-KGK-NH_2_; N-α-palmitoyl-l-lysyl-l-glycyl-l-lysine-amide, yield 80%, white solid. RP-HPLC retention factor* k* 7.07 (gradient B 2–98%/30 min); MS (MALDI-TOF) *m*/*z* 569.4 [M + H]^+^; MW (monois.) 568.4; FTIR: 3302, 2926, 2848, 1676, 1536 cm^−1^.

Lipopeptide 7: Palm-KGKG-NH_2_; *N*-α-palmitoyl-l-lysyl-l-glycyl-l-lysyl-glycine-amide, yield 75%, white solid. RP-HPLC retention factor* k* 7.10 (gradient B: 2–98%/30 min); MS (MALDI-TOF) *m*/*z* 626.5 [M + H]^+^; MW (monois.) 625.5; FTIR: 3298, 2928, 2856, 1672, 1538 cm^−1^.

Lipopeptide 8: Myr-K-NH_2_; N-α-myristoyl-l-lysine-amide, yield 80%, white solid. RP-HPLC retention factor* k* 7.22 (gradient B: 2–98%/30 min); MS (MALDI-TOF) *m*/*z* 356.4 [M + H]^+^; MW (monois.) 355.4; FTIR: 3302, 2919, 2851, 1674, 1536 cm^−1^.

Lipopeptide 9: Myr-KK-NH_2_; *N*-α-myristoyl-l-lysyl-l-lysine-amide, yield 82%, white solid. RP-HPLC retention factor* k* 6.33 (gradient B 2–98%/30 min); MS (MALDI-TOF) *m*/*z* 484.3 [M + H]^+^; MW (monois.) 483.3; FTIR: 3290, 2926, 2856, 1676, 1538 cm^−1^.

Lipopeptide 10: Myr-KKK-NH_2_; N-α-myristoyl-l-lysyl-l-lysyl-l-lysine-amide, yield 72%, white solid. RP-HPLC retention factor* k* 5.93 (gradient B 2–98%/30 min); MS (MALDI-TOF) *m*/*z* 612.5 [M + H]^+^; MW (monois.) 611.5; FTIR: 3287, 2926, 2853, 1677, 1538 cm^−1^.

Lipopeptide 11: Myr-KKKK-NH_2_; *N*-α-myristoyl-l-lysyl-l-lysyl-l-lysyl-l-lysine-amide, yield 78%, white solid. RP-HPLC retention factor* k* 5.57 (gradient B 2–98%/30 min); MS (MALDI-TOF) *m*/*z* 740.2 [M + H]^+^; MW (monois.) 739.2; FTIR: 3288, 2926, 2853, 1677, 1539 cm^−1^.

Lipopeptide 12: Myr-KG-NH_2_; *N*-α-myristoyl-*L*-lysyl-l-glycine-amide, yield 84%, white solid. RP-HPLC retention factor* k* 7.00 (gradient B 2–98%/30 min); MS (MALDI-TOF) *m*/*z* 413.4 [M + H]^+^; MW (monois.) 412.4; FTIR: 3290, 2919, 2851, 1679, 1541 cm^−1^.

Lipopeptide 13: Myr-KGK-NH_2_; N-α-myristoyl-l-lysyl-l-glycyl-l-lysine-amide, yield 74%, white solid. RP-HPLC retention factor* k* 6.25 (gradient B 2–98%/30 min); MS (MALDI-TOF) *m*/*z* 541.2 [M + H]^+^; MW (monois.) 540.2; FTIR: 3293, 2921, 2853, 1679, 1540 cm^−1^.

Lipopeptide 14: Myr-KGKG-NH_2_; *N*-α-myristoyl-l-lysyl-l-glycyl-l-lysyl-glycine-amide, yield 70%, white solid. RP-HPLC retention factor* k* 6.31 (gradient B 2–98%/30 min); MS (MALDI-TOF) *m*/*z* 598.4 [M + H]^+^; MW (monois.) 597.4; FTIR: 3290, 2923, 2854, 1632, 1543 cm^−1^.

**Fig. 2:**
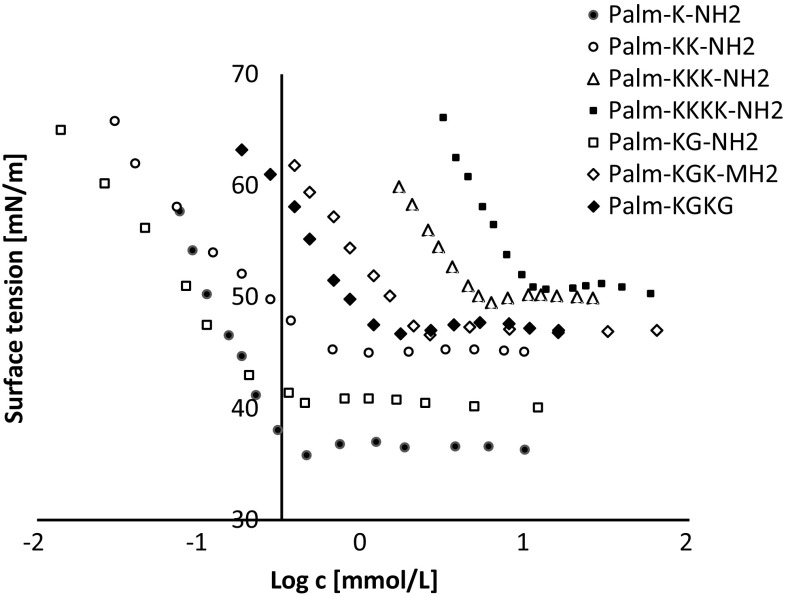
Surface tension vs. logarithm of concentration curves for the lipopeptide surfactants acylated with palmitic fatty acid

**Fig. 3:**
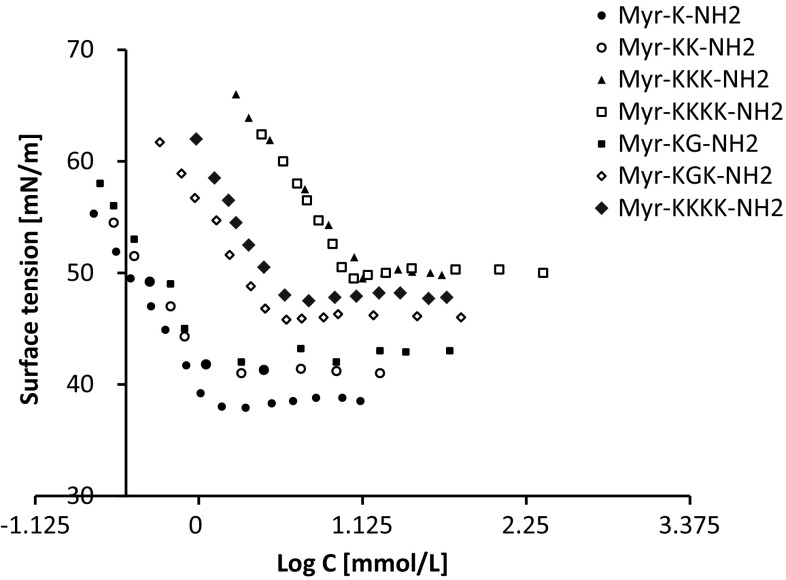
Surface tension vs. logarithm of concentration curves for the lipopeptide surfactants acylated with myristic fatty acid

**Fig. 4:**
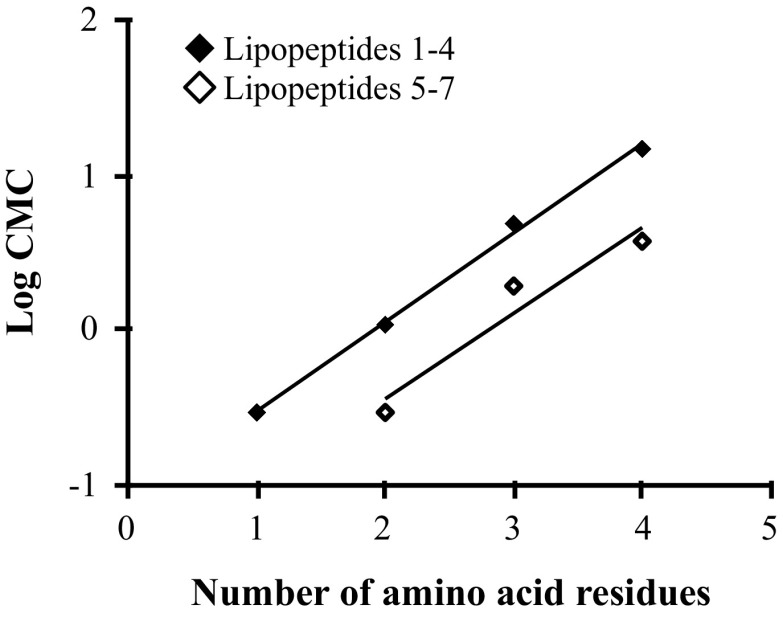
Dependence between CMC and the number of amino acid residues forming the hydrophilic head of lipopeptides acylated with palmitic fatty acid **1–4** (*filled diamond*) **5–7** (*open diamond*)

**Fig. 5:**
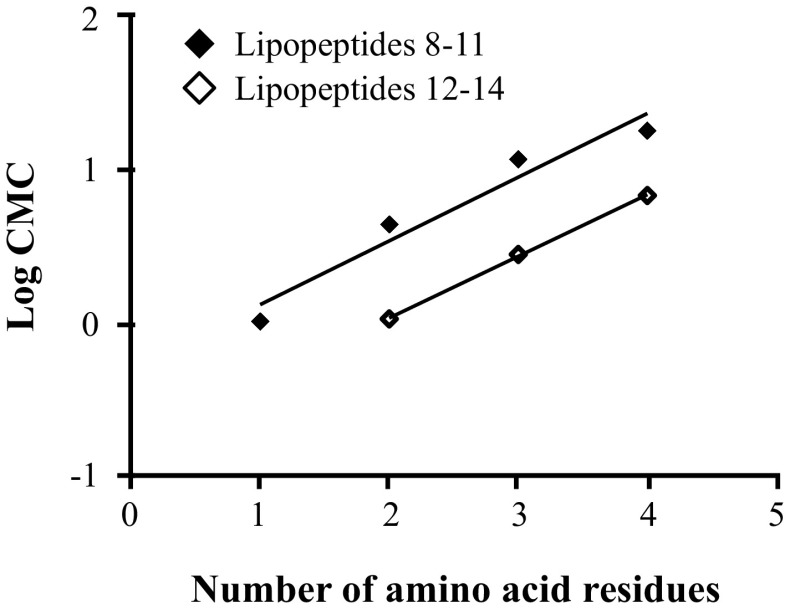
Dependence between CMC and the number of amino acid residues forming the hydrophilic head of lipopeptides acylated with myristic fatty acid **8–11** (*filled diamond*) and **12–14** (*open diamond*)

The surface tension properties of lipopeptides were established from the surface tension plots vs. logarithm of concentration of lipopeptides (Figs. 2, 3). The surface tension studies of water solutions of lipopeptides 1–7 indicated that the surface activity depends on the amino acid composition of the hydrophilic fragment. The lowest CMC value was obtained for compound Palm-K-NH_2_ (0.29 mmol/L). It was found that CMC increases with the amount of amino acids incorporated in the hydrophilic head of the lipopeptide and the highest CMC (14.6 mmol/L) was determined for Palm-KKKK-NH_2_. An identical relationship was observed for lipopeptides 8–14. The lowest CMC value was determined for Myr-K-NH_2_ (1.02 mmol/L) and the highest CMC value was recorded for Myr-KKKK-NH_2_ (18.04 mmol/L). Figures 4 and 5 show the linear relationship between Log CMC and the number of amino acids constituting the hydrophilic moiety of the surfactant within lipopeptides 1–7 and 8–14, respectively. It can be seen that CMC increases together with the increase in the number of amino acids. This tendency, observed for tested lipopeptides, occurs due to the fact that with increases in the size of the hydrophilic heads of the surfactants the tail becomes relatively shorter. Tadros indicates that for all ionic surfactants, CMC decreases with increasing alkyl chain length [21]. Brito et al. also found that for lipopeptide surfactants, where the hydrophilic fragment is a serine and tyrosine, CMC decreases when the length of the attached hydrophobic chain increases [29]. Perez et al. obtained methyl esters of ε-acylated lipopeptide surfactants where the hydrophilic fragment was a lysine residue and observed a linear decrease in CMC with an increasing hydrophobic portion of the molecule [30].

The *σ*_CMC_ parameter provides information about which value the surface tension is reduced to at a given CMC concentration. The *σ*_CMC_ has a smaller value when the *π*_CMC_ is larger, so that the system exhibits improved efficacy in reducing surface tension. For lipopeptides 1–7, the efficiency decreases (Table 1) the more amino acids they contain. The most effective is Palm-K-NH_2_, and this decreases the surface tension of water to 36.5 mN/m and the least effective is Palm-KKKK-NH_2_ (*σ*_CMC_ = 50.9 mN/m). The efficiency of reducing surface tension *σ*_CMC_ revealed for lipopeptides 8–14 (Table 2) also decreases with the increasing size of the hydrophilic fragment. Myr-K-NH_2_ (38.1 mN/m) most effectively lowers the surface tension of water, and Myr-KKKK-NH_2_ (50.4 mN/m) the least. The arginine-derivative surfactants, exemplified by LAE, present strong relationship between the surface tension at the CMC and the hydrophobic chain length [31]. This behavior of lipopeptides can be explained by the increasing hydrophilicity of the molecule. Considering the aqueous phase as a polar phase and air as the non-polar, more hydrophilic compounds will exhibit a stronger tendency to be “inside” the aqueous phase. Therefore, less hydrophilic molecules will be moved in the direction of the non-polar phase—the air; hence, their improved effectiveness in reducing surface tension. This is typical to various series of ionic and nonionic [32–36] surfactants. Better surface active properties have been described for a naturally produced lipopeptide surfactant—surfactin. It was found that surfactin can adopt a ball-like structure at the air/water interface; therefore it may be considered more as a hydrophobic nanoparticle than a typical surfactant. This spherical structure allows for tight packing of the molecules on the interface, and thus only a low concentration of surfactin (20 μM) is needed to significantly reduce the surface tension of water from 72 to 25 mN/m [37, 38].

Parameter pC_20_ , shown in Tables 1 and 2, allows the evaluation of the surface activity of a surfactant. It is defined as the negative log of the total concentration of surfactant required to reduce the surface tension by 20 mN/m. pC_20_ determined for lipopeptides **1–14** decreases when the number of amino acid residues increases. The highest value (pC_20_ = 3.95) was observed for Palm-KG-NH_2_, while the lowest (pC_20_ = 1.81) was noted for Myr-KKKK-NH_2_. This means that tested lipopeptides with smaller hydrophilic heads and longer hydrophobic tails have better ability to adsorb at the water/air interface, which is characteristic of all types of surface active agents [32–36].

**Fig. 6:**
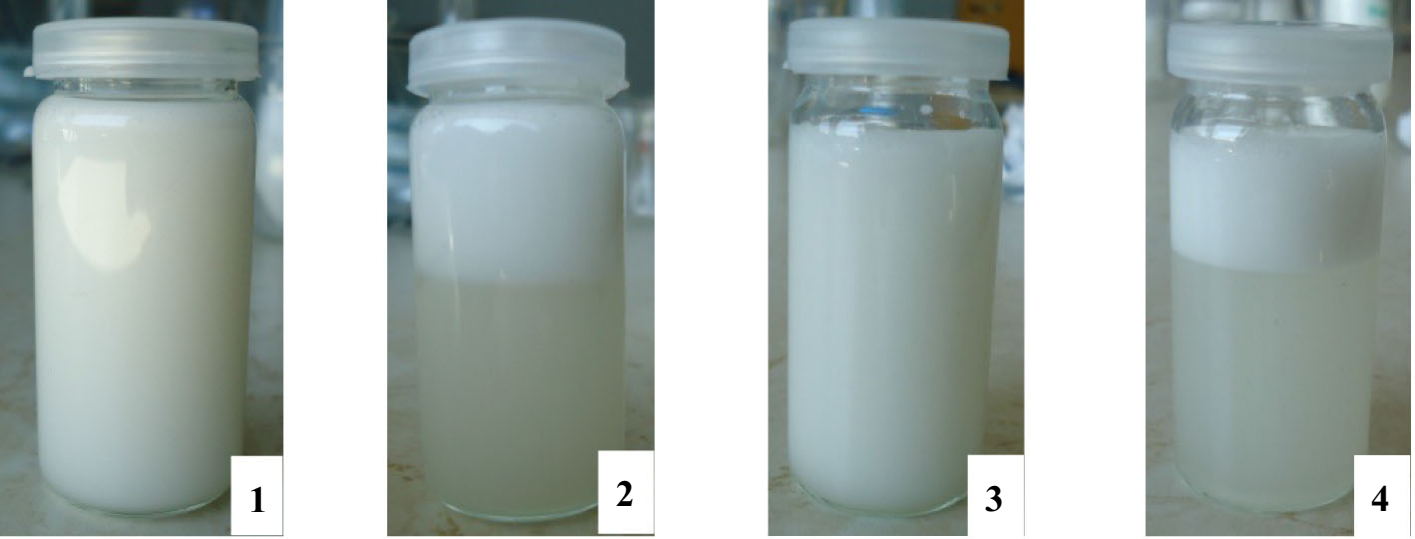
Emulsion consists of Miglyol 812 and water (*1*,* 2*) and paraffin oil and water (*3*,* 4*) stabilized with Palm-KK-NH_2_. Pictures were taken just after the homogenization process (*1*,* 3*) and 2 min after the homogenization process (*2*,* 4*)

Generally, stable emulsions are produced with use of emulsifiers, which are mixtures of homologues having hydrocarbon chains of different lengths. Mixtures of emulsifiers are able to form densely packed films at the oil/water interface that make them more flexible and more resistant to the emulsion ageing process. In this study, high purity lipopeptide surfactants were used to prepare emulsions. It was found that none of the lipopeptides created a stable emulsion (Tables 1, 2). All of the emulsions broke after less than 2 min. Examples of prepared emulsions are presented in Fig. 6. This proves that tested lipopeptides have weak emulsifying power and suggests that they cannot be used as emulsifiers. All of the studied lipopeptides have a net positive charge and behave like cationic surfactants, which, except for quaternary ammonium compounds, exhibit weak emulsification efficiency [39]. Nonetheless, it is possible to use them as auxiliary surfactants with disinfectant properties in combination with more potent emulsifiers.

## Conclusions

Fourteen cationic lipopeptide surfactants were synthesized and their physicochemical properties were characterized. It was established that, for lipopeptides, surface active properties depend on the length of the attached fatty acid chain as well as the composition of the hydrophilic head. Because lipopeptides are able to form micelles in aqueous solutions, it is possible that they may reveal solubilizing properties. It was found that lipopeptides do not show sufficient emulsification power to create stable paraffin oil/water or Miglyol 812/water emulsion systems. Considering their antimicrobial properties, which have been published elsewhere [19], and their ability to decrease the surface tension of water, lipopeptides might be used as preservatives and possibly as co-surfactants.

## References

[1] Randhawa KKS, Rahman PKSM (2014). Rhamnolipid biosurfactants-past, present, and future scenario of global market. Front Microbiol.

[2] Mnif I, Ghribi D (2016). Glycolipid biosurfactants: main properties and potential applications in agriculture and food industry. J Sci Food Agric.

[3] Kim P, Kim JH (2005). Characterization of a novel lipopolysaccharide biosurfactant from *Klebsiella oxitoca*. Biotechnol Bioprocess Eng.

[4] Mnif I, Ghribi D (2015). Review lipopeptides biosurfactants: mean classes and new insights for industrial, biomedical, and environmental applications. Biopolymers.

[5] Jha SS, Joshi SJ, Geetha SJ (2016). Lipopeptide production by *Bacillus subtilis* R1 and its possible applications. Braz J Microbiol.

[6] Baker SC, Chen C-Y (2010). Enrichment and purification of lipopeptide biosurfactants. Adv Exp Med Biol.

[7] Bezza FA, Chirwa EMN (2015). Production and applications of lipopeptide biosurfactant for bioremediation and oil recovery by *Bacillus subtilis* CN2. Biochem Eng J.

[8] Das K, Mukherjee AK (2007). Comparison of lipopeptide biosurfactants production by *Bacillus subtilis* strains in submerged and solid state fermentation systems using a cheap carbon source: some industrial applications of biosurfactants. Process Biochem.

[9] Vilhena C, Bettencourt A (2012). Daptomycin: a review of properties, clinical use, drug delivery and resistance. Mini Rev Med Chem.

[10] Humphries RM, Pollett S, Sakoulas G (2013). A current perspective on daptomycin for the clinical microbiologist. Clin Microbiol Rev.

[11] Zavascki AP, Goldani LZ, Li J, Nation RL (2007). Polymyxin B for the treatment of multidrug-resistant pathogens: a critical review. J Antimicrob Chemother.

[12] Yeh M-S, Wei Y-H, Chang J-S (2008). Enhanced production of surfactin from *Bacillus subtilis* by addition of solid carriers. Biotechnol Prog.

[13] Sen R (2010). Surfactin: biosynthesis, genetics and potential applications.

[14] Fernandes PAV, de Arruda IR, dos Santos AFAB (2007). Antimicrobial activity of surfactants produced by *Bacillus subtilis* R14 against multidrug-resistant bacteria. Braz J Microbiol.

[15] Sáenz-Marta CI, de Ballinas-Casarrubias LM, Rivera-Chavira BE, Nevárez-Moorillón GV (2015). Biosurfactants as useful tools in bioremediation. Adv Bioremediation Wastewater Polluted Soil.

[16] Zhang J, Xue Q, Gao H (2016). Production of lipopeptide biosurfactants by *Bacillus atrophaeus* 5-2a and their potential use in microbial enhanced oil recovery. Microb Cell Fact.

[17] Mani P, Sivakumar P, Balan SS (2016). Economic production and oil recovery efficiency of a lipopeptide biosurfactant from a novel marine bacterium *Bacillus simplex*. Achiev Life Sci.

[18] Scientific Committee on Consumer Safety (SCCS). Opinion on ethyl lauroyl arginate HCl. 2011. doi:10.2772/97780.

[19] Greber KE, Dawgul M, Kamysz W, Sawicki W (2017). Cationic net charge and counter ion type as antimicrobial activity determinant factors of short lipopeptides. Front Microbiol.

[20] Chan WC, White PD (2000). Fmoc solid phase peptide synthesis: a practical approach.

[21] Tadros T (2013). Critical micelle concentration. Encycl. colloid interface Science.

[22] Xu Q, Wang L, Xing F (2011). Synthesis and properties of dissymmetric gemini surfactants. J Surfactants Deterg.

[23] Aiad IA, Badawi AM, El-Sukkary MM (2012). Synthesis and biocidal activity of some naphthalene-based cationic surfactants. J Surfactants Deterg.

[24] Nagai A, Nagai Y, Qu H, Zhang S (2007). Dynamic behaviors of lipid-like self-assembling peptide A6D and A6K nanotubes. J Nanosci Nanotechnol.

[25] Giacometti A, Cirioni O, Kamysz W, Silvestri C, Del Prete MS, Licci A, D’Amato G, Łukasiak J, Scalise G (2005). In vitro activity and killing effect of citropin 1.1 against gram-positive pathogens causing skin and soft tissue infections. Antimicrob Agents Chemother.

[26] Sarig H, Rotem S, Ziserman L, Danino D, Mor A (2008). Impact of self-assembly properties on antibacterial activity of short acyl-lysine oligomers. Antimicrob Agents Chemother.

[27] Chen Y, Guarnieri MT, Vasil AI, Vasil ML, Mant CT, Hodges RS (2007). Role of peptide hydrophobicity in the mechanism of action of alpha-helical antimicrobial peptides. Antimicrob Agents Chemother.

[28] Jiang Z, Vasil AI, Hale JD, Hancock RE, Vasil ML, Hodges RS (2008). Effects of net charge and the number of positively charged residues on the biological activity of amphipathic alpha-helical cationic antimicrobial peptides. Biopolymers.

[29] Brito RO, Silva SG, Fernandes RMF (2011). Enhanced interfacial properties of novel amino acid-derived surfactants: effects of headgroup chemistry and of alkyl chain length and unsaturation. Colloids Surf B Biointerfaces.

[30] Pérez L, Pinazo A, Teresa García M (2009). Cationic surfactants from lysine: synthesis, micellization and biological evaluation. Eur J Med Chem.

[31] Singare PU, Mhatre JD (2012). Cationic surfactants from arginine: synthesis and physicochemical properties. Am J Chem.

[32] Infante MR, Perez L, Pinazo A. Novel cationic surfactants from arginine. In: Holmberg K, editor. Novel surfactants: preparation, application, and biodegradability. New York: Marcel Dekker; 1998.

[33] Anacker EW. Micelle formation of cationic surfactants in aqueous media. In: Jungermann E, editor. Cationic surfactants. New York: Marcel Dekker; 1970.

[34] Attwood D, Florence AT (1983). Surfactant systems: their chemistry, pharmacy, and biology.

[35] Rosen MJ (1978). Surfactants and interfacial phenomena.

[36] Varka EM, Coutouli-Argyropoulou E, Infante MR (2004). Synthesis, characterization, and surface properties of phenylalanine-glycerol ether surfactants. J Surfact Deterg.

[37] Shen HH, Thomas RK, Chen CY, Darton RC, Baker SC, Penfold J (2009). Aggregation of the naturally occurring lipopeptide, surfactin, at interfaces and in solution: an unusual type of surfactant?. Langmuir.

[38] Wang J, Jia D, Tao K, Wang C, Zhao X, Yaseen M, Xu H, Que G, Webster JR, Lu JR (2013). Interfacial assembly of lipopeptide surfactants on octyltrimethoxysilane-modified silica surface. Soft Matter.

[39] Troy DB, Beringer P (2006). Remington: the science and practice of pharmacy.

